# miR-199a Overexpression Enhances the Potency of Human Induced-Pluripotent Stem-Cell–Derived Cardiomyocytes for Myocardial Repair

**DOI:** 10.3389/fphar.2021.673621

**Published:** 2021-06-03

**Authors:** Weihua Bian, Wangping Chen, Thanh Nguyen, Yang Zhou, Jianyi Zhang

**Affiliations:** ^1^ Department of Biomedical Engineering, School of Medicine and School of Engineering, University of Alabama at Birmingham, Birmingham, AL, United States; ^2^ Informatics Institute, The University of Alabama at Birmingham, Birmingham, AL, United States; ^3^ Department of Medicine/Cardiovascular Diseases, The University of Alabama at Birmingham, Birmingham, AL, United States

**Keywords:** heart failure, cell cycle, microRNA, stem cells, myocardial infarction

## Abstract

Mammalian cardiomyocytes exit the cell cycle during the perinatal period, and although cardiomyocytes differentiated from human induced-pluripotent stem cells (hiPSC-CMs) are phenotypically immature, their intrinsic cell-cycle activity remains limited. Thus, neither endogenous cardiomyocytes nor the small number of transplanted hiPSC-CMs that are engrafted by infarcted hearts can remuscularize the myocardial scar. microRNAs are key regulators of cardiomyocyte proliferation, and when adeno-associated viruses coding for microRNA-199a (miR-199a) expression were injected directly into infarcted pig hearts, measures of cardiac function and fibrosis significantly improved, but the treatment was also associated with lethal arrhythmia. For the studies reported here, the same vector (AAV6-miR-199a) was transduced into hiPSC-CMs, and the cells were subsequently evaluated in a mouse model of myocardial infarction. AAV6-mediated miR-199a overexpression increased proliferation in both cultured and transplanted hiPSC-CMs, and measures of left ventricular ejection fraction, fractional shortening, and scar size were significantly better in mice treated with miR-199a–overexpressing hiPSC-CMs than with hiPSC-CMs that had been transduced with a control vector. Furthermore, although this investigation was not designed to characterize the safety of transplanted AAV6-miR-199a–transduced hiPSC-CMs, there was no evidence of sudden death. Collectively, these results support future investigations of miR-199a–overexpressing hiPSC-CMs in large animals.

## Introduction

Patients who recover from myocardial infarction (MI) frequently progress to heart failure, which is the leading cause of mortality worldwide. In adult mammals, cardiomyocytes exit the cell cycle shortly after birth (Porrello et al., 2011; Gao et al., 2018; Ye et al., 2018) and, consequently, the proliferation rate of endogenous cardiomyocytes is far too low to effectively repair the heart. Cardiac stem cells were once considered capable of differentiating into cardiomyocytes, but only rarely, and the evidence is somewhat controversial (Gunthel et al., 2018). Thus, techniques for reactivating the cell cycle in endogenous cardiomyocytes and/or repopulating the myocardial scar with exogenous cells continue to be among the most promising strategies for cardiac repair (Geng et al., 2018; Giacca 2020; Kino et al., 2020). Several studies have shown that microRNAs (miRNAs) are key regulators of cardiomyocyte proliferation (Pandey and Ahmed 2015; Qu et al., 2017; Huang et al., 2018; Fan et al., 2019; Hu et al., 2019; Wang et al., 2020; Li et al., 2021), and an extensive list of candidate human miRNAs have been identified *via* unbiased functional screening in mice (Eulalio et al., 2012; Braga et al., 2021). At least one of these candidates, miR-199a has been investigated in porcine models of myocardial injury (Gabisonia et al., 2019); the miRNA sequence was expressed from an adeno-associated virus (AAV) to prevent genomic integration, and the vector (AAV6-miR-199a) was delivered to infarcted hearts *via* direct intramyocardial injection. One month later, AAV6-miR-199a treatment was associated with marked improvements in cardiac function, muscle mass, and scar size, but persistent uncontrolled miR-199a expression eventually led to lethal arrhythmia in most of the animals.

For the studies reported here, the same vector was used to overexpress miR-199a in cardiomyocytes, and the cells were subsequently evaluated in a mouse MI model. The cardiomyocytes were differentiated from human induced pluripotent stem cells (hiPSCs), which can be used to generate a theoretically unlimited number of cells and are expected to be minimally immunogenic, because they can be reprogrammed from the somatic cells of each individual patient (Burridge et al., 2015; Richards et al., 2016; Pioner et al., 2019; Deicher and Seeger 2021). We hypothesized that this approach would reproduce many of the benefits associated with direct AAV6-miR-199a injection while substantially reducing the risk associated with long-term, unregulated miR-199a expression. Our results confirmed that AAV6-mediated miR-199a overexpression increased the proliferation of both cultured and transplanted hiPSC-derived cardiomyocytes (hiPSC-CMs) and that AAV6-miR-199a–transduced hiPSC-CMs significantly improved cardiac function and scar size, with no evidence of sudden death, after transplantation into infarcted mouse hearts.

## Materials and Methods

All protocols were approved by the Institutional Animal Care and Use Committee of the University of Alabama at Birmingham and were implemented in accordance with the Guidelines for the Care and Use of Laboratory Animals issued by the United States National Institutes of Health (2011).

### Human Induced-Pluripotent Stem Cells-Cardiomyocytes Differentiation and Purification

**FIGURE 1:**
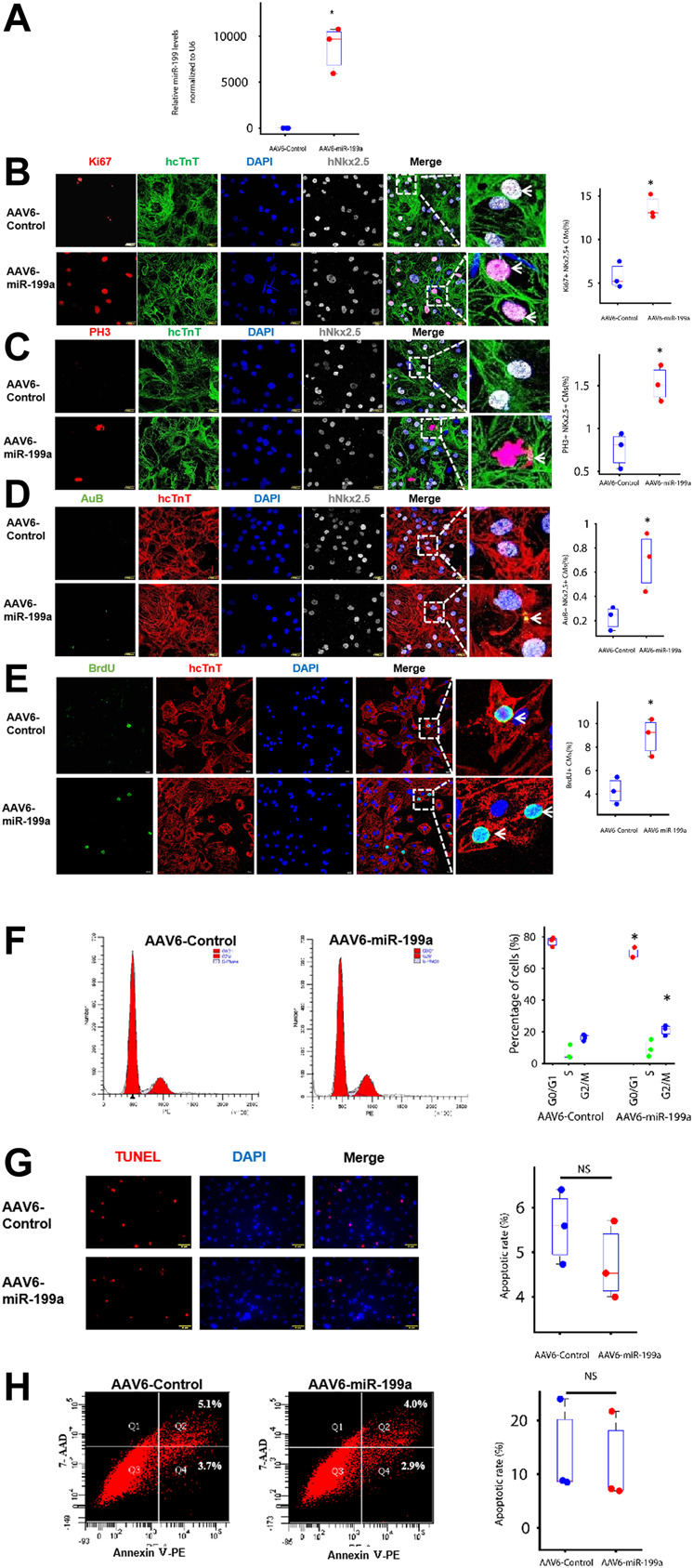
miR-199a overexpression upregulated the expression of markers for cell-cycle progression and proliferation in cultured hiPSC-CMs. hiPSC-CMs were transduced with AAV6 vectors coding for miR-199a (AAV6-miR-199a) or a control miR sequence (AAV6-Control). Ten days after transduction, the following experiments were performed. **(A)** miR-199a abundance in AAV6-miR-199a–and AAV6-Control–transduced hiPSC-CMs was compared *via* RT-qPCR. **(B–E)** AAV6-miR-199a and AAV6-Control hiPSC-CMs were stained for expression of the human isoform of cardiac troponin T (hcTnT) and for expression of **(B)** the proliferation marker Ki67, the M-phase markers **(C)** phosphorylated histone 3 (PH3), **(D)** Aurora B (AuB) and **(E)** the S-phase marker Brdu. Nuclei were identified by staining with DAPI and for Nkx2.5 expression; then, the number of cells that expressed each marker was quantified and presented as a percentage of the total number of cardiomyocytes. **(F)** hiPSC-CMs were stained with a quantitative DNA dye; then, the amount of DNA in each cell was measured *via* flow cytometry and used to determine the proportion of cells in the G0/G1, S, and G2/M phases of the cell cycle. **(G)** Apoptotic hiPSC-CMs were identified by TUNEL staining, and nuclei were counterstained with DAPI; then, apoptosis was quantified as the percentage of TUNEL-positive cells. **(H)** Apoptotic cells were identified by staining for the presence of Annexin V; then, the cells were evaluated *via* flow cytometry, and apoptosis was quantified as the percentage Annexin-V–positive cells. *n* = 3 independent experiments, 3 wells per experiment, and 5 images per well. *p < 0.05 vs. AAV6-Control; NS, not significant.

**FIGURE 2:**
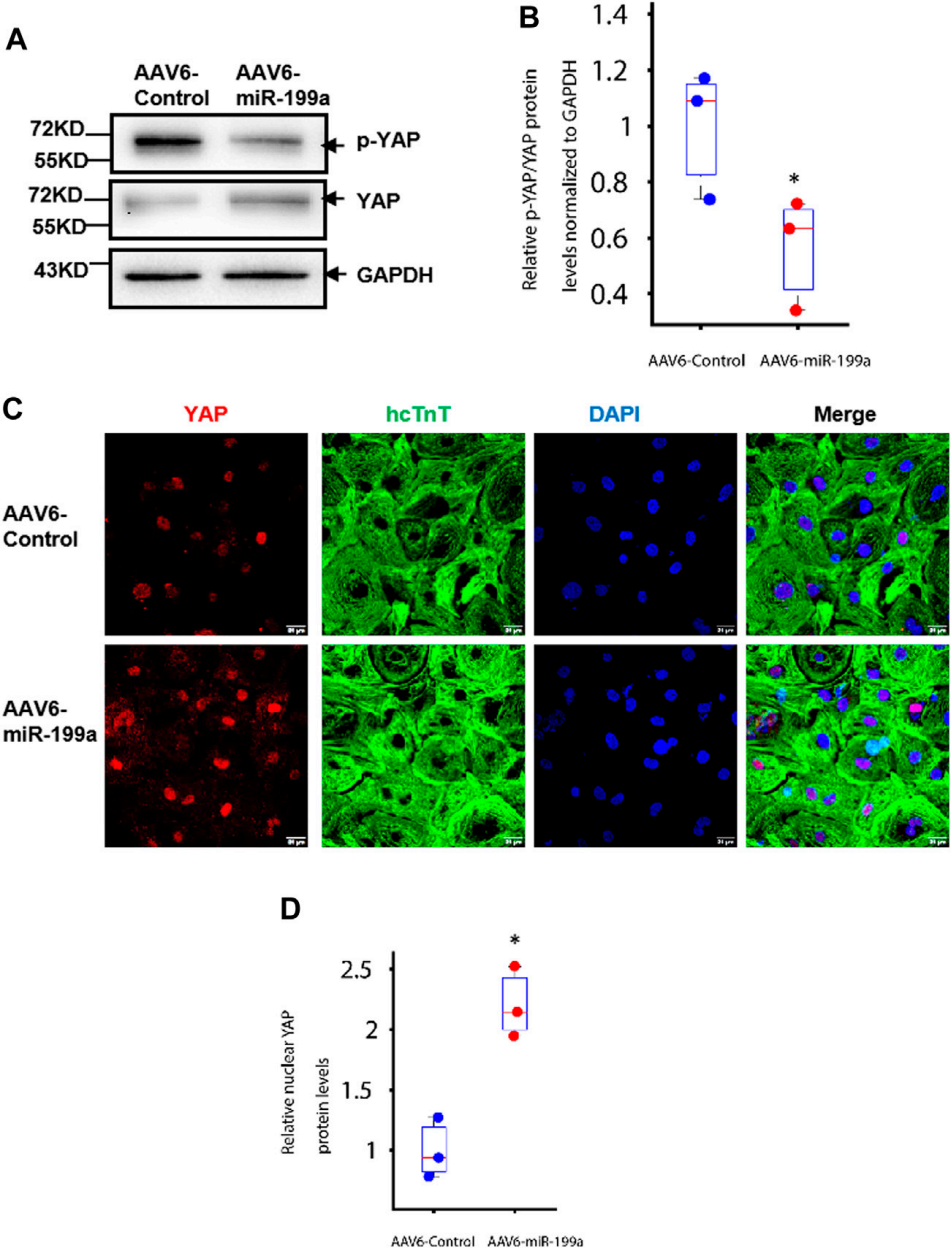
miR-199a overexpression reduced YAP phosphorylation and increased nuclear YAP abundance in hiPSC-CMs. hiPSC-CMs were transduced with AAV6-miR-199a or AAV6-Control. Ten days after transduction, the following experiments were performed. **(A–B)** YAP and phosphorylated YAP (p-YAP) protein abundance was **(A)** evaluated *via* Western blot, quantified *via* enhanced chemiluminescence, and **(B)** presented as the ratio of p-YAP to YAP. **(C–D)** hiPSC-CMs were **(C)** stained for the presence of YAP and hcTnT, nuclei were labeled with DAPI, and **(D)** YAP protein in the nucleus of AAV6-miR-199a was quantified by the fluorescence density of YAP-positive nuclei in AAV6-miR-199a normalized to that in the AAV6-Control group. *n* = 3 independent experiments, 3 wells per experiment, and 5 images per well. *p < 0.05 vs. AAV6-Control.

hiPSC-CMs were differentiated from a noncommercial line of hiPSCs that was generated in our laboratory as previously described in details (Zhang et al., 2015). The hiPSCs were reprogrammed from cardiac fibroblasts that had been isolated from the left atrium of an adult male patient with heart failure who had no known genetic disorders. Reprogramming was performed by transfecting the cardiac fibroblasts with Sendai virus encoding OCT4, SOX2, KLF4 and c-myc, and the hiPSCs were established from clone #5. The generation and complete characterization of this hiPSC line (hiPSC#5), including assessments of pluripotency, surface marker expression, karyotype, and the PSC cells’ evidently unlimited capacity for self-renewal, has been published previously (Zhang et al., 2015); (Zhang et al., Circ Heart Fail, 8 (2015) 156–166; Figures 1, 2). For hiPSC-CMs differentiation, hiPSCs were seeded in 6-well plates with mTeSR medium (STEMCELL Technologies), and the medium was changed daily. The cells were passaged three times and then cultured until 90% confluent before differentiation. Differentiation was induced by culturing the hiPSCs in RPMI 1,640 medium with 2% B27 minus insulin (RB-medium; Gibco), 10 μM CHIR99021 (STEMCELL Technologies), and 1 μg/ml insulin (Sigma) for 24 h; in RB-medium containing 2.5 μM CHIR99021 for 48 h; in RB medium containing 10 μM IWR1 (STEMCELL Technologies) for 48 h; and in RB medium for 48 h; then, the medium was replaced with 1,640 medium supplemented with 2% B27 supplement (Gibco). Differentiated hiPSC-CMs were purified *via* metabolic selection in glucose-free RPMI medium (Gibco) containing B27 supplement (Gibco) and 4 mM lactate (Sigma) for 5 days. hiPSC-CM identity was confirmed *via* the observation of beating cells, which typically appear ∼7 days after the differentiation protocol is initiated, and by analysis of the expression of cardiomyocyte markers (e.g., human cardiac troponin T [hcTnT]) (Zhu et al., 2018).

### Production and Transduction of Recombinant Adeno-Associated Virus6 Viral Particles

All plasmids used to generate the miR-199a and Control adeno-associated virus vectors (AAV6-miR-199a and AAV6-Control, respectively), were kindly provided by Dr. Mauro Giacca (International Centre for Genetic Engineering and Biotechnology, Italy). AAV6-miR-199a and AAV6-Control viruses were packaged in 293AAV cells, viral stocks were obtained with an AAVpro^®^ Purification Kit (Takara), and titration of the AAV particles was performed with an AAVpro^®^ Titration Kit (Takara). hiPSC-CM transduction was conducted 17 days after differentiation was initiated with a multiplicity of infection (MOI) of 1,000. Briefly, the hiPSC-CMs were cultured to 80% confluence, and the medium was replaced with fresh medium containing the AAV6-miR-199a or AAV6-Control. The cells were incubated at 37°C for 12 h, and then the medium was replaced with 1,640 medium supplemented with 2% B27 supplement. miR-199a expression was analyzed *via* real-time quantitative polymerase chain reaction (RT-qPCR).

### Real-Time Quantitative Polymerase Chain Reaction

Total RNA was extracted with an miRNeasy Mini Kit (Qiagen) as directed by the manufacturer’s instructions, quantified by Nanodrop, and converted into cDNA with a miRCURY LNA RT kit (Qiagen). qPCR was performed with a miRCURY LNA SYBR Green PCR Kit (Qiagen), and miR-199a amplification was performed with primers from the hsa-miR-199a miRCURY LNA miRNA PCR Assay (Qiagen). U6 snRNA miRCURY LNA miRNA PCR Assay (Qiagen) was used for normalization, and the relative fold change of miR-199a was determined *via* the 2^−ΔΔCT^ method.

### Immunofluorescence Analyses

10 days after AAV6-Control or AAV6-miR-199a transduction, cells were immobilized with 4% paraformaldehyde (PFA) for 10 min, permeabilized with acetone for 1 min, washed with phosphate-buffered saline (PBS) + 0.1% Tween 20 (PBST), blocked with 10% donkey serum (Life Technologies, Carlsbad, CA) for 30 min, and incubated at 4°C overnight with primary antibodies against Ki67, phosphorylated histone 3 (PH3), Aurora-B (AuB), Yes-associated protein (YAP), phosphorylated YAP (p-YAP), hcTnT, and/or human Nkx2.5 (Supplementary Table S1). Then, the cells were incubated for 1 h with fluorophore-linked secondary antibodies, and nuclei were stained with 4′,6-diamidino-2-phenylindole (DAPI) at room temperature for 10 min. Imaging was performed with a confocal microscope.

Hearts of mice were dehydrated with 30% sucrose, cut into 10-μm sections, washed with PBST for 10 min, fixed with 4% PFA for 10 min, permeabilized with cold acetone for 3 min, blocked with 10% donkey serum for 30 min, and incubated at 4°C overnight with antibodies against Ki67, PH3, hcTnT, and/or human Nkx2.5; then, the sections were incubated for 1 h with fluorophore-linked secondary antibodies, and nuclei were stained with DAPI at room temperature for 10 min. Sections were sealed with Vectashield Antifade Mounting Medium (Vector Laboratories) and photographed with a confocal microscope.

### Engraftment

Because the hiPSCs were derived from humans, hiPSC-CM engraftment was evaluated *via* immunofluorescence analysis of hcTnT expression. Hearts were embedded in OCT compound, frozen, and cut into sections at 10-μm intervals from the apex to base. hcTnT-expressing cells were counted in every twentieth section of the whole heart; then, the total was multiplied by 20 to determine the number of hiPSC-CMs in the entire heart, divided by the number of cells administered (3 × 10^5^), and reported as a percentage.

### Cell Cycle Analysis *via* Cellular DNA Quantification

Cell cycle analysis was performed as described previously (Li et al., 2020). Briefly, hiPSC-CMs were digested, washed with PBS, resuspended in 75% ethanol at 4°C overnight, washed with PBS, resuspended in propidium iodide (PI) staining solution (50 μg/ml PI in PBS with 0.1% Triton and 100 μg/ml RNase A) and incubated for 30 min at room temperature; then, PI fluorescence, which is proportional to DNA content, was monitored with a flow cytometer (BD Bioscience) to determine the cell-cycle phase for each cell. Data were analyzed with FlowJo software.

### Annexin V-Phycoerythrin/7-Aminoactinomycin D Assay

hiPSC-CMs were cultured in six-well plates, digested, washed with PBS, and resuspended in 100 μL of binding buffer; then, 5 μL of 7-Aminoactinomycin D (7-AAD) solution and 5 μL of phycoerythrin-conjugated Annexin V solution were added, and the cells were incubated for 15 min before the addition of 400 µL binding buffer. Apoptosis (i.e., the proportion of Annexin-V–positive cells) was evaluated with a flow cytometer.

### Terminal Deoxynucleotidyl Transferase (dUTP) Nick-End Labeling

TUNEL was performed with a TUNEL Assay Kit (Roche, Basel, Switzerland). Briefly, cultured hiPSC-CMs or frozen sections were fixed with 4% PFA, permeabilized with PBS containing 0.1% Triton X-100 and 0.1% sodium citrate, washed with PBST, stained with TUNEL solution for 1 h at 37°C, washed with PBST, and blocked with 10% donkey serum (Life Technologies, Carlsbad, CA) for 30 min; frozen sections were also incubated with hcTnT at 4°C overnight after TUNEL-staining was completed. Cells and sections were incubated with fluorophore-linked secondary antibodies at room temperature for 1 h, and nuclei were labeled with DAPI; then, hiPSC-CM apoptosis was evaluated by determining the proportion of cells (cultured hiPSC-CMs) or hcTnT-positive cells (frozen sections) that were TUNEL-positive.

### Western Blotting

hiPSC-CMs were lysed with M-PER™ Mammalian Protein Extraction Reagent (Fisher Scientific); then, proteins were separated in 4–20% Precast Protein Gels (Bio-Rad) and transferred to a polyvinylidene fluoride (PVDF) membrane. The membranes were blocked with 5% nonfat dry milk for 1 h, incubated overnight at 4 °C with primary antibodies against YAP, p-YAP, and glyceraldehyde phosphate dehydrogenase (GAPDH), and then with secondary antibodies at room temperature for 1 h (Supplementary Table S1). Proteins on the membrane were detected with an enhanced chemiluminescence reagent kit (Millipore) and quantified with ImageJ software.

### Mouse Myocardial Infarction (MI) Model and Human Induced-Pluripotent Stem Cells-Cardiomyocytes Transplantation

NOD/SCID Gamma mice (stock #005557; The Jackson Laboratory) were anesthetized with inhaled isoflurane (1.5–2%), intubated, and ventilated; then, the left thoracic cavity was opened, and the left anterior descending (LAD) coronary artery was ligated. The infarcted region was identified by blanching of the myocardium, and the border zone was identified by striations of red at the edges of the blanched region. The AAV6-miR-199a–transduced hiPSC-CMs or AAV6-Control–transduced hiPSC-CMs (suspended in PBS) were intramyocardially injected into three sites (1 × 10^5^ cells in 5 μL medium/site, 3 × 10^5^ cells in 15 μL medium/animal); one site was located in the infarcted zone, and the other two were located in the border zone of the infarct. After treatment was completed, the chest was closed, and animals were intraperitoneally injected with buprenorphine (0.1 mg/kg) every 12 h for three consecutive days, and with carprofen (5 mg/kg) every 12 h for one day after surgery. Control assessments were conducted in animals administered equivalent injections of cell-free PBS after MI and in animals that underwent all surgical procedures for MI induction except ligation of the LAD coronary artery (i.e., Sham surgery). Experiments were conducted in seven animals per treatment group.

### Echocardiography

Echocardiography was performed as described previously (Fan et al., 2020). Mice were mildly anesthetized with 1.5–2% isoflurane, ensuring that the heart rate was maintained at 400–500 beats per minute; then, a Micro-Ultrasound system (Vevo 2100, VisualSonics Inc.) was used to obtain both parasternal long-axis (B-mode) and short-axis (M-mode) echocardiographic data, and the B-mode data were used to guide orientation of the M-mode data. The images were analyzed with Vevo software to determine the ventricular ejection fraction (EF) and fractional shortening (FS); results obtained from analyses of the long-axis and short-axis data were equivalent.

### Infarct Size

Infarct size was assessed as described previously (Zhu et al., 2018). Briefly, hearts were embedded in OCT compound, frozen, and cut into sections at 1.0-mm intervals from the apex to base; then, five sections from each heart were fixed in Bouin’s solution and stained with Sirius red to identify the myocardial scar and with fast green to identify functional myocardium. Sections were photographed, and the ratio of the scar area to the total area of the left ventricle was calculated with Image J software (Kong et al., 2018).

### Statistical Analysis

Statistical analyses were performed with Matlab software (version 2020a). Because the data were collected from a small number of samples, analyses were conducted *via* nonparametric statistics, and our results were presented as box plots. Comparisons between two groups with *n* > 3 were evaluated *via* the Wilcoxon-ranksum test (Mann and Whitney 1947), and comparisons among three or more groups were evaluated *via* Kruskal-wallis test (Kruskal and Wallis 1952). *p* < 0.05 was considered statistically significant.

## Results

### miR-199a Overexpression Activated the Cell Cycle in Cultured hiPSC-CMs

To avoid genomic integration, miR-199a overexpression was induced *via* transduction with adeno-associated virus coding for miR-199a (AAV6-miR-199a). hiPSC-CMs were transduced 10 days after completion of the differentiation protocol (Zhu et al., 2018), and miR-199a overexpression was confirmed 10 days after AAV6-miR-199a transduction *via* RT-qPCR: miR-199a was 1,000-fold more abundant in hiPSC-CMs transduced with AAV6-miR-199a than in hiPSC-CMs transduced with the control AAV6 vector (AAV6-Control) (Figure 1A). Cells expressing markers for proliferation (Ki67) and for phase M (phosphorylated histone 3 [pH3] and Aurora B [AuB]) and phase S (bromodeoxyuridine [BrdU] incorporation) of the cell cycle were ∼2-fold more common after transduction with AAV6-miR-199a than with AAV6-Control (Figures 1B–E), while assessments of DNA content indicated that the percentage of G0/G1-phase cells was significantly lower among AAV6-miR-199a hiPSC-CMs (69.3%) than AAV6-Control hiPSC-CMs (77.0%, *p* < 0.05). AAV6-miR-199a transduction also tended to increase the proportion of S phase and G2/M phase cells, and the percentage of G2/M-phase cells between two groups was significant (Figure 1F), and assessments of apoptosis (TUNEL assay, Annexin V expression) (Figures 1G, H) did not differ significantly between the two cell groups. Thus, miR-199a overexpression promoted hiPSC-CM proliferation and cell-cycle progression without significantly altering measures of apoptosis or necrosis.

### miR-199a Overexpression Activated the Yes-Associated Protein Signaling Pathway in hiPSC-CMs

Experiments in neonatal rat cardiomyocytes have shown that miR-199a directly targets the YAP inhibitory kinase TAOK1 and the E3 ubiquitin ligase β-TrCP, thereby impeding YAP degradation and promoting the translocation of YAP into the nucleus (Torrini et al., 2019). Furthermore, observations in mice suggest that increases in YAP abundance and nuclear localization are sufficient to induce the proliferation of cardiac cells and to improve cardiac performance after MI (Flinn, Link, and O'Meara 2020). Here, we found that the ratio of Serine-127–phosphorated YAP (p-YAP) to total YAP abundance was significantly lower (Figures 2A, B), while nuclear YAP levels were significantly greater, in AAV6-miR-199a–than in AAV6-Control–transduced hiPSC-CMs. (Figures 2C, D). The relative YAP protein level in the nucleus of AAV6-miR-199a was quantified by the fluorescence density of YAP-positive nuclei in AAV6-miR-199a and normalized to that in the AAV6-Control group. Thus, miR-199a overexpression likely reduced the ubiquitin-mediated proteasomal degradation of YAP and increased nuclear YAP abundance, which may have contributed to the enhanced proliferation and cell-cycle activity observed in AAV6-miR-199a–transduced hiPSC-CMs.

### miR-199a Overexpression Enhanced the Potency of hiPSC-CMs for Myocardial Recovery in a Mouse MI Model

**FIGURE 3:**
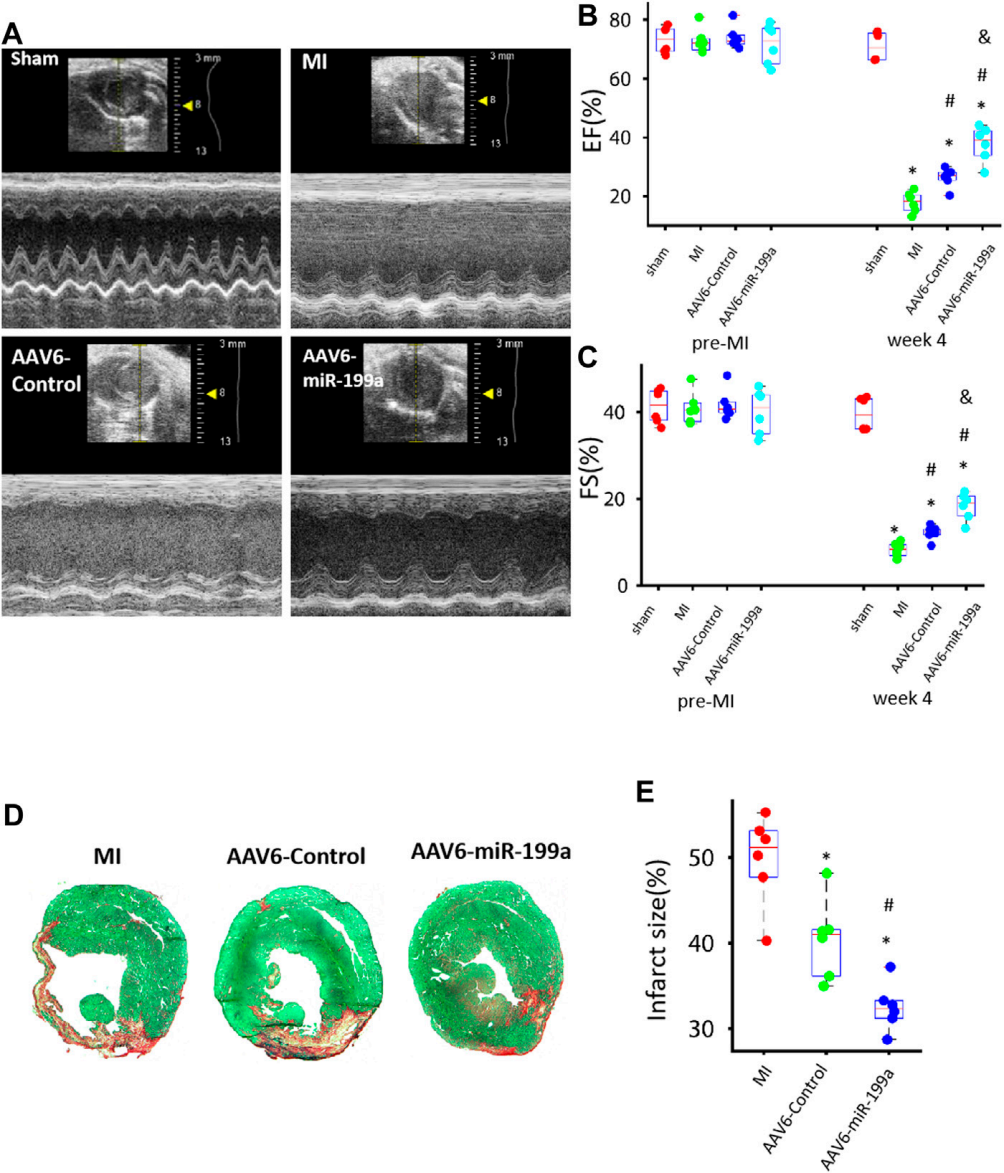
AAV6miR-199a–tranduced hiPSC-CMs were more potent than AAV6-Control–transduced hiPSC-CMs for myocardial recovery in a mouse MI model. MI was induced in mice *via* LAD coronary artery ligation, and then the animals were treated with AAV6-miR-199a–transduced hiPSC-CMs (the AAV6-miR-199a group), AAV6-Control–transduced hiPSC-CMs (the AAV6-Control group), or PBS (the MI group); Sham animals underwent all surgical procedures for MI induction except the ligation step and recovered without either experimental treatment. Treatments were administered *via* intramyocardial injection at one site in the infarcted region and two sites in the border zone of the infarct; 1 × 10^5^ cells were administered per injection site (3 × 10^5^ cells/animal). **(A)** Echocardiographic images were collected before MI (or Sham) surgery (pre-MI) and 4 weeks afterward (week 4) and evaluated for calculations of left-ventricular **(B)** ejection fraction (EF) and **(C)** fractional shortening (FS). *p < 0.05 vs. Sham, ^#^ *p* < 0.05 vs. MI, ^&^ *p* < 0.05 vs. AAV6-Control; *n* = 6 animals per group. **(D–E)** Left ventricular sections from hearts explanted 4 weeks after MI induction were **(D)** stained with Sirius red and fast green; functional myocardium appears green and scar tissue appears red. **(E)** Infarct size was calculated as the ratio of scar area to the total area of the left ventricular surface. *p < 0.05 vs. MI, ^#^ *p* < 0.05 vs. AAV6-Control; *n* = 6 per group.

Whether miR-199a overexpression could enhance the potency of hiPSC-CMs for myocardial recovery was evaluated in a mouse MI model. MI was induced *via* ligation of the LAD coronary artery; then, animals in the AAV6-miR-199a group were treated with AAV6-miR-199a–transduced hiPSC-CMs, animals in the AAV6-Control group were treated with AAV6-Control–transduced hiPSC-CMs, and animals in the MI group were treated with an equivalent volume of PBS. A fourth group of animals, the Sham group, underwent all surgical procedures for MI induction except LAD artery ligation and recovered without either experimental treatment. Three animals died during treatment administration, and the overall survival rate was 85% at Week 4. Echocardiographic assessments (Figure 3A) of left-ventricular ejection fraction (EF, Figure 3B) and fractional shortening (FS, Figure 3C) were conducted both before and 4 weeks after MI induction and cell administration; then, the animals were sacrificed, and infarct sizes were determined in Sirius-red–and fast-green–stained sections from the left ventricle (Figures 3D, E). All three parameters (EF, FS, and infarct size) were significantly better in both hiPSC-CM treatment groups than in MI animals, and in AAV6-miR-199a animals than in AAV6-Control animals, at Week 4.

### miR-199a Overexpression Increased the Number of Engrafted and Proliferating hiPSC-CMs in Infarcted Mouse Hearts

**FIGURE 4:**
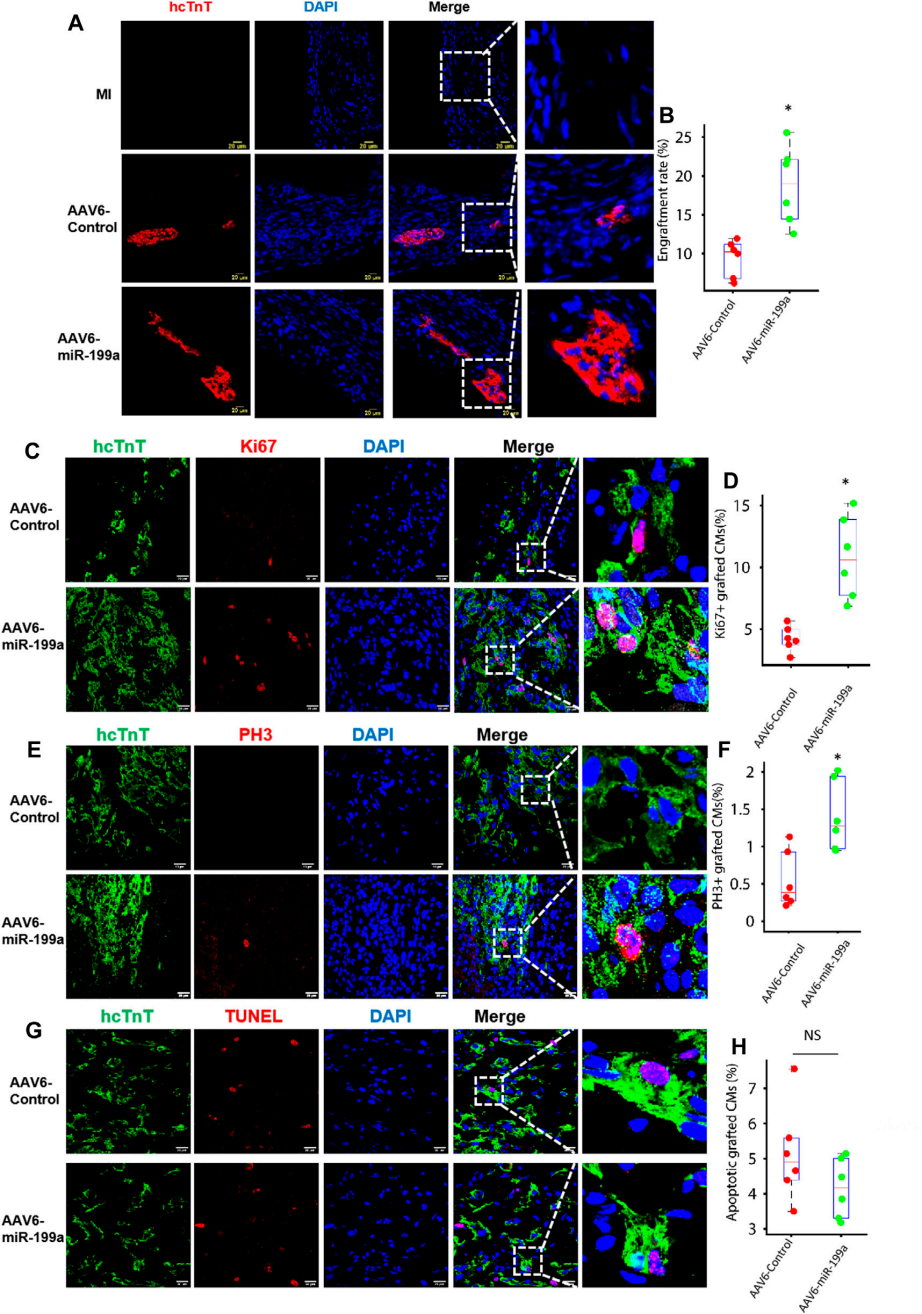
The number of engrafted and proliferating hiPSC-CMs was greater in the infarcted hearts of mice treated with AAV6-miR-199a–transduced than with AAV6-Control–transduced hiPSC-CMs. Four weeks after MI induction, hearts from animals in the AAV6-miR-199a and AAV6-Control groups were embedded in OCT compound, frozen, and cut into sections at 10-μm intervals from the apex to base. **(A)** Sections were stained with anti-hcTnT antibodies to identify transplanted hiPSC-CMs, and nuclei were labeled with DAPI; then, **(B)** the ratio of the number of hcTnT-positive cells to the total number of cells administered was calculated and presented as a percentage. **(C–F)** Engrafted hiPSC-CMs were identified *via* hcTnT expression, **(C)** proliferating cells were identified *via* Ki67 expression, **(D)** cells in phase M of the cell cycle were identified by the presence of PH3, and nuclei were labeled with DAPI; then, hiPSC-CM **(E)** proliferation and **(F)** cell-cycle activity was quantified as the percentage of hcTnT-expressing cells that also expressed Ki67 and PH3, respectively. **(G)** Engrafted hiPSC-CMs were identified *via* hcTnT expression, apoptotic cells were identified by TUNEL staining, and nuclei were labeled with DAPI; then, **(H)** hiPSC-CM apoptosis was quantified as the percentage of hcTnT-expressing cells that were positive for TUNEL. Quantified results were presented as mean ± SD. *p < 0.05 vs. AAV6-Control. Five randomly selected fields were evaluated in each of five sections from the border zone of infarction per animal; *n* = 6 animals per group.

The fundamental goal of cell-based cardiac therapies is to replace damaged myocardium with functional cardiac muscle; thus, since some evidence suggests that stem-cell–derived cardiomyocytes can become electromechanically integrated with the native myocardium after transplantation into infarcted mammalian hearts (Shiba et al., 2012; Chong et al., 2014; Shiba et al., 2016), we investigated whether the enhanced potency of miR-199a–overexpressing hiPSC-CMs for myocardial recovery was accompanied by an increase in the number, proliferation, and cell-cycle activity of engrafted cells. Four weeks after MI induction and treatment, hiPSC-CMs were identified in sections from the hearts of AAV6-miR-199a and AAV6-Control animals by expression of the human isoform of cardiac troponin T (hcTnT), proliferation was evaluated *via* Ki67 expression, and cell-cycle progression was evaluated *via* PH3 expression. When expressed as a percentage of the total number of cells administered, engrafted hiPSC-CMs were approximately twice as common in hearts from AAV6-miR-199a animals than from AAV6-Control animals (Figures 4A, B), and a significantly greater proportion of engrafted AAV6-miR-199a–transduced hiPSC-CMs than engrafted AAV6-Control–transduced hiPSC-CMs expressed Ki67 or PH3 (Figures 4C–F). Notably, the proportion of TUNEL-positive hiPSC-CMs in the hearts of animals from both groups did not differ significantly (Figures 4G, H). Collectively, these results are fully consistent with our observations in cultured cells and suggest that the enhanced engraftment of AAV6-miR-199a-transduced hiPSC-CMs was at least partially attributable to an increase in the proliferation of engrafted cells.

## Discussion

miRNAs are crucially involved in numerous cellular processes, and as many as 96 have been at least tentatively linked to the regulation of DNA synthesis and cytokinesis in hiPSC-CMs (Diez-Cunado et al., 2018). Direct intramyocardial injections of AAV vectors coding for miR-199a overexpression significantly improved cardiac performance and fibrosis when evaluated in a porcine model of myocardial injury (Gabisonia et al., 2019); however, most of the animals died from lethal arrhythmias that were likely caused by persistent and uncontrolled miR-199a overexpression. Here, we show that many of the benefits associated with direct intramyocardial AAV6-miR-199a administration can also be induced *via* the transplantation of miR-199a-overexpressing hiPSC-CMs: AAV6-mediated miR-199a overexpression increased hiPSC-CM proliferation both in culture and after transplantation into infarcted mouse hearts, and measures of left ventricular EF, FS, and scar size were significantly better in mice treated with miR-199a–overexpressing hiPSC-CMs than with hiPSC-CMs in which miR-199a expression had not been manipulated. Notably, miR-199a overexpression was also associated with declines in YAP phosphorylation and increases in nuclear YAP abundance, which is consistent with previous reports that most of the miRNAs implicated in hiPSC-CM proliferation target components of the Hippo signaling pathway (Diez-Cunado et al., 2018). Furthermore, although this investigation was not designed to thoroughly characterize the safety concerns associated with the administration of AAV6-miR-199a–transduced hiPSC-CMs, we found no evidence of sudden death in treated animals.

Early cell-based approaches for the repair of injured heart tissue often involved the administration of bone marrow-derived cells, cardiac progenitor cells, or mesenchymal stem cells; however, improvements in myocardial recovery were only mild and are believed to be induced primarily *via* the cells’ paracrine activity. Researchers have also begun to develop techniques for transdifferentiating fibroblasts directly into cardiomyocytes (Sadahiro 2019), which suggests that endogenous cardiac fibroblasts could be a viable target for regenerative myocardial therapy, but the efficiency of the transdifferentiation procedure is exceptionally low. Thus, the transplantation of cardiomyocytes differentiated from human embryonic stem cells or, especially, hiPSCs (Romagnuolo et al., 2019; Sadahiro 2019; Yoshida et al., 2020) continues to be among the most promising strategies for regenerative myocardial therapy (Yoshida and Yamanaka 2017; Samak and Hinkel 2019; Weber et al., 2020). Nevertheless, despite substantial evidence that hiPSC-CMs tend to more closely resemble fetal cardiomyocytes than the cardiomyocytes of adult animals (Robertson, Tran, and George 2013), their intrinsic cell-cycle activity remains limited and, consequently, the small number of transplanted hiPSC-CMs that are typically retained and survive at the site of administration cannot substantially remuscularize the myocardial scar (Isomi, Sadahiro, and Ieda 2019). When hiPSC-CMs that had been engineered to overexpress the cell-cycle regulator CCND2 were administered to infarcted mouse hearts, the number of engrafted cells approximately tripled over a three week period (Zhu et al., 2018), and hiPSC-CMs occupied ∼50% of the scarred region by month 6 (Fan et al., 2019). However, since CCND2 was stably overexpressed, the transplanted cells could (at least in theory) proliferate indefinitely. Thus, AAV6-miR-199a–transduced hiPSC-CMs may be associated with fewer long-term safety concerns, because miR-199a expression is upregulated only transiently. miR-199a–overexpressing hiPSC-CMs may also be less immunogenic than intramyocardially administered AAV6-miR-199a (Barnes et al., 2019), but longer-term studies are needed to more thoroughly characterize the safety of this treatment strategy.

In conclusion, this study demonstrates that AAV6-miR-199a–tranduced hiPSC-CMs can reproduce many of the benefits observed when direct intramyocardial injections of AAV-miR-199a were evaluated in a porcine MI model, but with no evidence of sudden death. Collectively, these results support future investigations of this treatment strategy in large-animal models, particularly for the evaluation of arrhythmogenic complications, which have been observed in pigs and non-human primates, but are rarely reported in rodents.

## Data Availability

The raw data supporting the conclusions of this article will be made available by the authors, without undue reservation.
