# Supplementary material for: miR-199a Overexpression Enhances the Potency of Human Induced-Pluripotent Stem-Cell–Derived Cardiomyocytes for Myocardial Repair
Source: Front Pharmacol. 2021 Jun 3;12:673621. doi: 10.3389/fphar.2021.673621 (PMC8209326; doi:10.3389/fphar.2021.673621)
Supplement: Supplementary file 1 [file Table1.DOCX]

**Supplemental Table I. Antibodies.**

| Antigen | Manufacturer | Catalog# | Type | Immunohistochemistry | Western  blot |
| --- | --- | --- | --- | --- | --- |
| Human Cardiac  Troponin T | Abcam | ab91605 | Rabbit monoclonal | 1:300 |  |
| Human Cardiac  Troponin T | R&D Systems | MAB1874 | Mouse Monoclonal | 1:100 |  |
| Ki67 | Abcam | ab16667 | Rabbit monoclonal | 1:100 |  |
| Aurora B | BD Biosciences | 611082 | Mouse Monoclonal | 1:50 |  |
| Phosphorylated  Histone H3 | EMD Millipore | 06-570 | Rabbit polyclonal | 1:1000 |  |
| YAP | Cell Signaling | 14074 | Rabbit Monoclonal | 1:100 | 1:1000 |
| Phospho-YAP | Cell Signaling | 13008 | Rabbit Monoclonal |  | 1:1000 |
| GAPDH | Abcam | ab22555 | Rabbit polyclonal |  | 1:1000 |
| Fitc-donkey  anti-mouse | Jackson  ImmunoResearch  Laboratory | 715-095-150 | Polyclonal | 1:300 |  |
| Cy™3  -donkey anti-rabbit | Jackson  ImmunoResearch  Laboratory | 711-165-152 | Polyclonal | 1:300 |  |
| Cy5-donkey  anti-mouse | Jackson  ImmunoResearch  Laboratory | 715-175-150 | Polyclonal | 1:300 |  |
| Anti-Rabbit IgG–Peroxidase antibody | Sigma-Aldrich | A0545 | polyclonal |  | 1:5000 |
